# Environmental Exposure to Cyanobacteria Hepatotoxins in a Pacific Island Community: A Cross-Sectional Assessment

**DOI:** 10.3390/microorganisms10081607

**Published:** 2022-08-09

**Authors:** Brenda Y. Hernandez, Jason Biggs, Xuemei Zhu, Patrick Sotto, Michelle Nagata, Ana Joy Pacilan Mendez, Yvette Paulino

**Affiliations:** 1 University of Hawaii Cancer Center, 701 Ilalo Street, Honolulu, HI 96813, USA; 2 University of Guam Cancer Research Center, Mangilao, GU 96913, USA

**Keywords:** cyanobacteria, cyanotoxins, hepatotoxins, microcystin, nodularin, cylindrospermopsin, anabaenopeptin

## Abstract

(1) Background: Cyanobacteria produce a wide range of secondary metabolites, including tumor-promoting hepatotoxins. We recently reported evidence of an independent association between oral cyanobacteria and hepatocellular carcinoma in a U.S. population. We sought to characterize the nature, sources, and health correlates of cyanotoxin exposure in the U.S. Pacific Island territory of Guam, which has a high incidence of liver cancer. (2) Methods: Seventy-four adult males and females were enrolled in a cross-sectional study to quantify cyanotoxins in saliva, urine, and blood and their correlation with health behaviors, medical history, and environmental exposures. Plant samples were collected from locations throughout the island. Microcystin/nodularin (MC/NOD), cylindrospermopsin (CYN), and anabaenopeptin (AB) were measured in biospecimens and in plant extracts by ELISA. (3) Results: Overall, among study participants MC/NOD were detected in 53.9% of saliva, 7.5% of urine, and 100% of serum.; CYN in 40.0% of saliva, 100.0% of urine, and 70.4% of serum; AB in 30.8% of saliva, 85% of urine, and 92.6% of serum. Salivary MC/NOD levels were significantly higher in individuals using municipal tap water as their primary source of drinking water; both salivary and urinary MC/NOD levels were higher in those not using store-bought/commercial water. Urine MC/NOD levels were highest among individuals consuming fruits and vegetables exclusively from local sources. Urine MC/NOD levels were elevated in individuals with hypertension and hyperlipidemia and salivary MC/NOD in those with recent alcohol consumption. Cyanotoxins were prevalent in plant samples including MC/NOD (46.6%), CYN (35.1%), and AB (51.7%). (4) Conclusions: Our study provides evidence that exposure to cyanobacterial hepatotoxins, including tumor promoters, may be prevalent in Guam and may originate from environmental sources. Population-based epidemiologic studies are needed to investigate the role of cyanotoxins in liver cancer development.

## 1. Background

Cyanobacteria are photosynthetic, gram-negative bacteria residing in nearly all terrestrial and aquatic environments across the world [1]. Among the diverse array of secondary metabolites produced by cyanobacteria are toxins targeting the nervous system, skin, gastrointestinal tract, and liver. A number of cyanotoxins affecting the liver are tumor-promoting and potentially carcinogenic, including microcystin (MC), nodularin (NOD), and cylindrospermopsin (CYN) [2,3,4,5,6]. MC are widespread cyclic peptides produced by numerous cyanobacteria species, including of the genera *Aphanizomenon*, *Dolichospermum* (also known as *Anabaena*), *Fischerella*, *Microcystis*, *Nostoc*, *Oscillatoria*, *Planktothrix*, *Synechococcus*, and *Trichodesmium* [7]. MC include approximately 250 congeners of which the highly toxic, MC-leucine arginine (MC-LR), is the most common and best characterized [7]. MC are considered to be tumor promoters and possible human carcinogens, primarily acting through the binding to and inhibition of protein serine/threonine phosphatases, PP1 and PP2A, which induces hyperphosphorylation of intracellular proteins and abnormal signaling in multiple pathways leading to cytoskeleton alterations, lipid peroxidation, oxidative stress, apoptosis, impaired DNA repair, and cellular proliferation [2,3]. NOD, a cyclic peptide structurally similar to MC, are produced by the genera *Nodularia* and *Nostoc* and include ten variants [4,8]. NOD also acts through the inhibition of PP1 and PP2A and shares some of the tumor-promoting properties of MC [4]. CYN are alkaloids produced by the genera *Raphidiopsis* (also known as *Cylindrospermopsis*), *Umezakia*, *Aphanizomenon*, Dolichospermum (*Anabaena*), *Lyngbya*, *Oscillatoria*, *Phormidium*, and *Planktothrix* [5]. In the liver, CYN has been shown to be cytotoxic, genotoxic, and potentially carcinogenic and inhibits the synthesis of protein and glutathione, an endogenous antioxidant, leading to oxidative stress and DNA strand breaks [5,6]. AB, which are comparatively not well-characterized, are cyclic peptides produced by the genera *Anabaena*, *Planktothrix*, *Nodularia*, and *Microcystis* and include over 96 congeners [9,10]. Some ABs inhibit PP1 and PP2A while others inhibit serine protease or exopeptidases [9,10].

Exposure to cyanotoxins occurs though oral ingestion of contaminated drinking water and food sources as well as inhalation of aerosolized toxin and dermal contact [11]. The potential for cyanotoxin exposure has escalated with rising global temperatures combined with eutrophication of water bodies leading to cyanobacteria overgrowth, typically manifesting as visible blooms [1,12]. Nonetheless, the nature, sources, and health consequences of cyanotoxin exposure in human populations are poorly understood. Few studies have directly evaluated the role of cyanobacteria and cyanotoxins in liver cancer development. Zheng et al. observed an independent association of serum MC levels with risk of hepatocellular carcinoma (HCC) in a case-control study in southwest China [13]. In a case-control study in a northeast U.S. population, we found an independent association of oral cyanobacteria with risk of HCC [14].

A high burden of liver cancer is found in Micronesia, including the U.S. Pacific Island territory of Guam where incidence rates are particularly elevated among the indigenous CHamoru population [15,16]. We previously detected cyanobacteria and MC, NOD, CYN, and AB in oral samples of residents of Guam [17], intimating a possible link to liver cancer. In the present study we sought to characterize the nature, sources, and health correlates of exposure to cyanobacteria hepatotoxins in Guam residents.

## 2. Methods

The Institutional Review Boards of the University of the Hawaii and the University of Guam approved this investigation and written informed consent was obtained from all study participants. The study was promoted through social, print, and broadcast media. Seventy-three adult residents of Guam ages 20–64 years old were enrolled in the cross-sectional study in June 2019–February 2020. (Study enrollment was terminated before enrollment goals were met due to COVID-19 restrictions.) Study participants were interviewed by study staff using structured questionnaires addressing health behaviors, medical conditions, sources of drinking water and food, and recreational water activity. Height, weight, waist, and blood pressure were measured. Saliva, blood, and first-catch urine were collected in order to obtain localized and systemic measures of cyanotoxins. In consideration of personal and cultural sensitivities, participants were allowed to select the types of biospecimens to donate of the 73 study participants, saliva was collected from 65 participants, blood from 54, and first-catch urine from 40.

Saliva samples were washed with 1× phosphate buffered saline (PBS), vortexed, and the supernatant transferred to a separate tube. Whole blood samples were collected using tubes containing no anti-coagulant, kept at room temperature for 30 min to allow clotting, and centrifuged at 2800 RPM for 10 min after which and the serum was separated from the clot and transferred to separate tubes. Urine samples were centrifuged and the supernatant transferred to new tubes. Processed samples were temporarily stored at –20 °C in the Guam laboratory and shipped on ice for testing to the University of Hawaii Cancer Center in Honolulu, Hawaii.

*Areca catechu* nuts and *Piper betle* leaves samples were collected using disposable gloves and individual specimen bags. These plants were selected as they are widely available and commonly used for Areca nut (AN)/betel quid (BQ) chewing, a common practice in Guam as well as other regions of the Pacific and Asia [18,19]. Plant samples were collected from sources throughout Guam and included 14 *Areca catechu* nuts and 14 *Piper betle* leaves. Eight *Areca catechu* and 9 *Piper betle* plants grown in Yap (Federated States of Micronesia) and sold at stores in Guam were also obtained. Local plants with similar morphologies were also collected including 2 samples of the invasive vine *Antigonon leptopus* and 3 samples of the invasive palm, *Livistona chinensis.* Plant samples were transferred on ice to the University of Guam study laboratory where they were separately freeze dried and shipped to Honolulu for testing at the UH Cancer Center. The outer husks were removed from Areca nuts and prepared for testing. (Nut samples, which previously yielded high background, were not tested.) Husk and leaf samples were chopped using sterile blades, vortexed in 1 mL of 1 × PBS, and refrigerated overnight at 4 °C. Following vortexing and centrifugation, the supernatant containing aqueous plant extracts were removed for testing.

All samples (serum, saliva, urine, and plant extracts) were subjected to three cycles of freezing at –80 °C followed by thawing in order to lyse cyanobacterial cells and facilitate the measurement of both free and cell-bound cyanotoxins in samples. Samples were tested using direct competitive enzyme-linked immunosorbent assays (ELISA) for MC/NOD, CYN, and AB (Eurofins Abraxis, Westminister, PA, USA). MC and NOD were evaluated in the same assay due to cross-reactivity of the structurally similar analytes. The sensitivity was ≥100% for microcystin congeners -LR and -LW and 66–92% for -LA, -RR, -LF, -YR, and -LY while sensitivity for nodularin was 78%. The CYN assay had documented cross-reactivity for related compounds deoxy- and 7-epi CYN. The AB assay covered congeners AA, B, F, and 872. The minimum detection level of each assay was 0.10 ng/mL for MC/NOD, 0.04 ng/mL for CYN, and 0.08 ng/mL for AB.

Assay samples were read at 450 nm on a BioTek ELx808 microplate reader (BioTek Instruments, Inc., Winooski, VT, USA). Samples, positive controls, and standards with known analyte concentrations were each tested in duplicate (50–100 uL/well) and mean absorbance values calculated. Sample analyte concentration was derived relative to the standard curve generated from the values of the standard samples.

The statistical analysis focused on the comparison of cyanotoxin levels by sample type and by participant and plant characteristics. Cyanotoxin levels were evaluated as both categorical variables (positive/negative, based on the minimum assay detection level) and continuous variables (regardless of minimum assay detection level). Statistical tests included chi-square, Wilcoxon rank sum, generalized linear models, and Spearman and Pearson correlation. The statistical significance threshold was <0.05 for all tests. SAS version 9.4 (SAS Institute, Carey, NC, USA) was used for statistical analyses.

## 3. Results

In the study population of 73 Guam residents aged 20–64 years (mean 34.8, std. dev. 13.7), 53% were female, 41% of CHamoru ancestry, and 65% were single/never married (Table 1). Fifteen percent were current cigarette smokers and 62% had consumed alcohol within the prior 30 days. Sixty-one percent of individuals were obese (≥30 kg/m^2^). Thirty percent (19/64) of participants reported a history of hypertension and 40% (28/70) had measured hypertension at the time of the study visit, defined as systolic ≥130 mm Hg and/or diastolic ≥80–89 mm Hg. A history of high cholesterol was reported by 20%, type 2 diabetes by 9%, and non-alcoholic fatty liver disease (NAFLD) by 3%.

Over half of individuals were current chewers of AN/BQ and 10 were former chewers. Thirty-four of the 35 current AN/BQ chewers had used AN/BQ for 10 years or longer. Among current chewers, 11 chewed the *Areca* nut only and 24 chewed quid, which included both nut and *Piper betle* leaf. Among the 24 current quid chewers, all prepared their quid with slaked lime (calcium hydroxide); 9 also added alcohol and 13 added tobacco. Of current chewers, 9 acquired AN/BQ from locally-grown sources, 6 purchased them from stores (AN/BQ imported from Yap), and 18 obtained them from both locally-grown and store-bought sources.

Primary drinking water sources reported by individuals were not mutually exclusive and included 79% using store-bought/bottled water (with or without other sources), 16% using home tap water—all of which were faucet-filtered (with or without other sources)- and 5% using rain water (with or without other sources). Sources of fresh vegetables included local sources only (9%), imported sources only (37%), and both local and imported sources (54%). Sources of fresh fruits included local sources only (9%), imported sources only (35%), and both local and imported sources (56%). Consumption of ocean seafood was reported as daily (3%, 2/66), sometimes (59%), and never or rarely (38%). Freshwater food consumption was reported as daily (2%), sometimes (42%), and never or rarely (56%).

Overall, MC/NOD was detected in 46.6% (27/58) of plant samples (mean 0.73 ng/mL, standard deviation (SD) 1.41, range 0–5.0), CYN in 35.1% (20/57) (mean 0.04 ng/mL, SD 0.07, range 0–0.24), and AB in 51.7% (30/58) (mean 0.28 ng/mL, SD 0.53, range 0–2.0). Levels of MC/NOD and AB were strongly correlated across plant samples (r = 0.81; *p* < 0.0001). *Antigonon leptopus* samples contained higher mean levels of MC/NOD (mean 5.0 ng/mL, SD 0) and AB (mean 2.0 ng/mL, SD 0 ng/mL) compared to MC/NOD and AB levels in *Piper betle* (mean 0.75 ng/mL, SD 0.51 and mean 0.26 mg/mL, SD 0.14), Areca husk (mean 0.52 ng/mL, SD 1.55 and mean 0.22 ng/mL, SD 0.57), and *Livistona chinensis* (mean 0.03 ng/mL, SD 0.05 and mean 0 ng/mL) (*p* = 0.0001 and <0.0001, respectively). *Piper betle* samples had the highest levels of CYN (mean 0.10 ng/mL, SD 0.07) compared to Areca husk (mean 0.002 ng/mL, SD 0.008), *Antigonon leptopus* (mean 0 ng/mL), and *Livistona chinensis* (mean 0.02 ng/mL, SD 0.02) (*p* < 0.0001). Among Guam plant samples, cyanotoxin levels did not vary across north, central, or southern regions of the island. Compared to Guam AN/BQ samples, Yap AN/BQ samples had higher levels of CYN (mean 0.04 ng/mL, SD 0.05 vs. mean 0.09 ng/mL, SD 0.09, *p* = 0.04) and higher levels of AB (mean 0.13 ng/mL, SD 0.18 vs. mean 0.38 ng/mL, SD 0.53, *p* = 0.04). MC/NOD levels did not vary between Guam and Yap samples.

**Figure 1:**
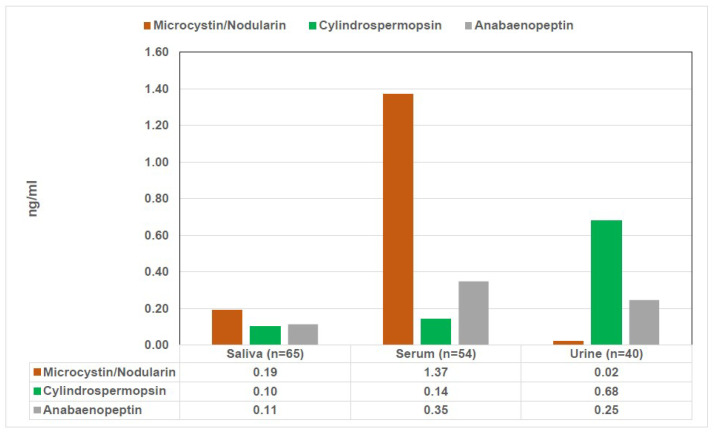
Cyanotoxin levels in human biospecimens.

Overall, MC/NOD was detected in 53.9% (35/65) of saliva samples (mean 0.19 ng/mL, SD 0.62, range 0–5.0), 7.5% (3/40) of urine samples (mean 0.02 ng/mL, SD 0.09, range 0–0.45), and 100% (54/54) of serum samples (mean 1.37 ng/mL, SD 0.75, range 0.35–3.8) (Figure 1). CYN was found in 40.0% (26/65) of saliva (mean 0.10 ng/mL, SD 0.10, range 0–0.82), 100.0% (40/40) of urine (mean 0.68 ng/mL, SD 0.38, range 0.13–2.0), and 70.4% (38/54) of serum samples (mean 0.14 ng/mL, SD 0.09, range 0–0.39). AB were detected in 30.8% (20/65) of saliva (mean 0.11 ng/mL, SD 0.25, range 0–2.0), 85% (34/40) of urine (mean 0.25 ng/mL, SD 0.13, range 0–0.52), and 92.6% (50/54) of serum samples (mean 0.35 ng/mL, SD 0.17, range 0–0.70).

Levels of each cyanotoxin did not correlate across saliva, urine, and serum with the exception of salivary and serum levels of CYN, which were moderately correlated (r = 0.36; *p* = 0.01). Overall, serum levels of all cyanotoxins were strongly correlated with one another (r = 0.74–0.87; *p* < 0.0001). Salivary levels of MC/NOD and AB were very strongly correlated (r = 0.94; *p* < 0.0001). Urinary levels of CYN and AB were strongly correlated (r = 0.66; *p* < 0.0001). Serum levels of MC/NOD were moderately correlated with salivary CYN (r = 0.39; *p* = 0.005). Serum AB and urinary CYN levels were inversely correlated (r = −0.54; *p* = 0.002).

Salivary AB was more prevalent (14/31, 45.2% vs. 6/34, 17.7%; *p* = 0.02) and detected at higher levels (mean 0.17 ng/mL, SD 0.35 vs. mean 0.06 ng/mL, SD 0.04; *p* = 0.003) in individuals 30 years and older compared to those <30 years, respectively. Salivary AB was also detected at higher levels in individuals residing in Guam for 20 years or longer compared to <20 years (mean 0.16 ng/mL, SD 0.34 vs. mean 0.06 ng/mL, SD 0.05; *p* = 0.009). Conversely, serum AB was detected at lower levels in individuals residing in Guam for 20 years or longer (mean 0.29 ng/mL, SD 0.18) relative to those living <20 years (mean 0.41 ng/mL, SD 0.14) (*p* = 0.018). Salivary AB was more prevalent and detected at higher levels among current AN/BQ chewers who used *Piper betle* leaf compared to current chewers who did not (12/22, 54.6% vs. 1/8, 12.5%; *p* = 0.04) (mean 0.20 ng/mL, SD 0.41 vs. mean 0.06 ng/mL, SD 0.05; *p* = 0.03).

**Figure 2:**
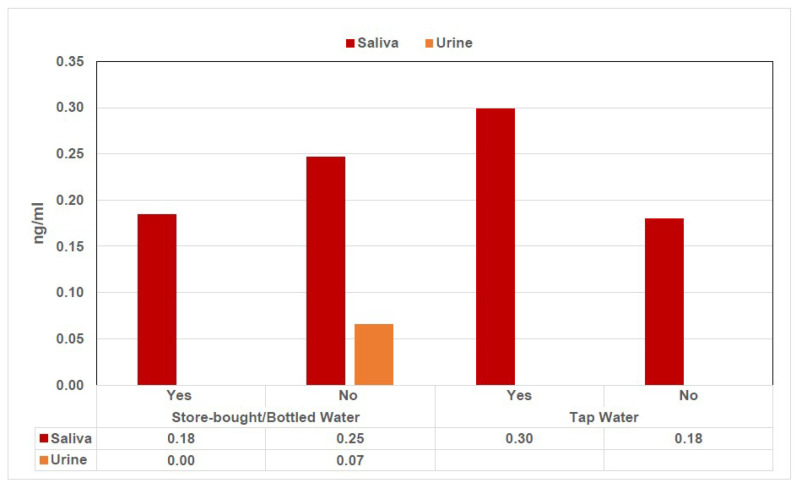
Elevated (*p* < 0.05) microcystin/nodularin levels in saliva and urine by drinking water source. Sources not mutually exclusive; Store-bought/Bottled: saliva (yes, *n* = 48; no, *n* = 13); urine (yes, *n* = 22; no, *n* = 14); Tap water: saliva (yes, *n* = 9; no, *n* = 52).

Individuals using home tap water as the primary source of drinking water had a higher prevalence and higher levels of salivary MC/NOD compared to those not drinking tap water (8/9, 88.9% vs. 25/52, 48.1%; *p* = 0.02) (mean 0.30 ng/mL, SD 0.26 vs. mean 0.18 ng/mL, SD 0.69; *p* = 0.0003) (Figure 2). Compared to those using store-bought/commercial water as the primary source of drinking water (exclusively or along with other sources), individuals not using store-bought/commercial water sources had a higher prevalence and higher levels of both salivary MC/NOD (22/48, 45.8% vs. 11/13, 84.6%; *p* = 0.013) (mean 0.18 ng/mL, SD 0.72 vs. mean 0.25 ng/mL, SD 0.23; *p* = 0.01) and urinary MC/NOD (3/14, 21.4% vs. 0/22, 0%; *p* = 0.02) (mean 0 ng/mL vs. mean 0.07 ng/mL, SD 0.14; *p* = 0.03).

**Figure 3:**
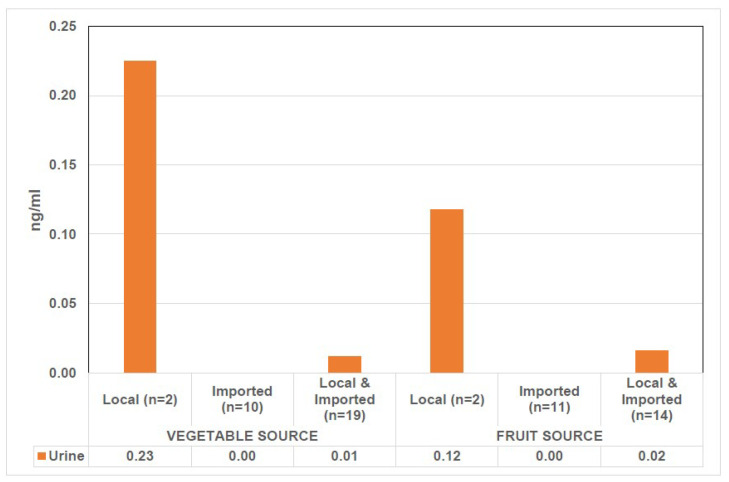
Elevated (*p* < 0.05) microcystin/nodularin levels urine by vegetable and fruit source.

Urine MC/NOD was most prevalent and demonstrated the highest levels among individuals consuming vegetables from local sources (1/2, 50%) (mean 0.23 ng/mL, SD 0.32) compared to those consuming vegetables from imported sources (0/10) (mean 0 ng/mL) or both local and imported sources (1/19, 5.3%) (mean 0.01 ng/mL, SD 0.05) (*p* = 0.03; *p* = 0.0014) (Figure 3). Similarly, urine MC/NOD was most prevalent and levels highest among individuals consuming fruits from local sources (1/2, 50%) (mean 0.12 ng/mL, SD 0.17) compared to those consuming vegetables from imported sources (0/11) (mean 0 ng/mL) or both local and imported source (1/14, 7.1%) (mean 0.02 ng/mL, SD 0.06) (*p* = 0.05; *p* = 0.04).

Urine AB was more prevalent and showed higher levels among individuals consuming vegetables from imported and both local and imported sources (9/10, 90%, mean 0.27 ng/mL, SD 0.11 and 17/19, 89.5%, mean 0.25 ng/mL, SD 0.11, respectively) compared to no detection in two individuals consuming vegetables from local sources only (*p* = 0.02). Serum AB levels varied by participation in river and ocean recreational activities with higher levels in those with no or only occasional participation (mean 0.30 ng/mL, SD 0.15 and 0.42 ng/mL, SD 18, respectively) compared to those with monthly or more frequent participation (mean 0.25 ng/mL, SD 0.18) (*p* = 0.02).

**Figure 4:**
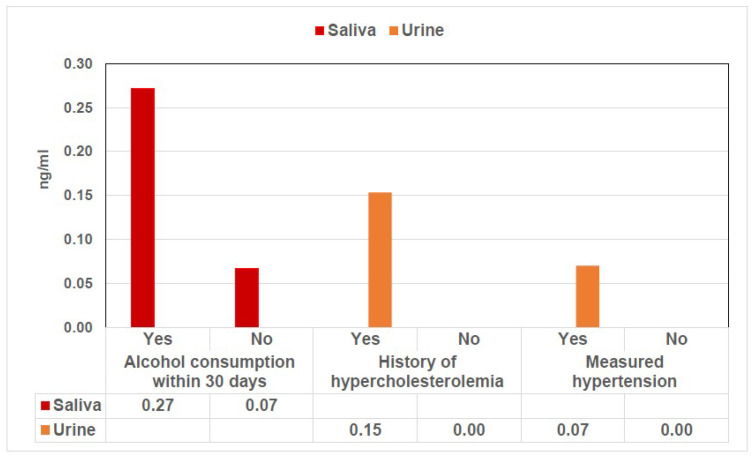
Elevated (*p* < 0.05) microcystin/nodularin levels in saliva and urine by liver cancer risk factors. Alcohol consumption within 30 days (yes, *n* = 37; no, *n* = 23); History of hypercholesterolemia (yes, *n* = 3; no, *n* = 29); Measured hypertension: (yes, *n* = 13; no, *n* = 26).

Cyanotoxin detection also varied by certain health conditions. Salivary AB was more prevalent and showed higher levels among individuals with a history of hypertension compared to those with no history (9/15, 60% vs. 10/41, 24.4%; *p* = 0.01) (mean 0.26 ng/mL, SD 0.49 vs. mean 0.07 ng/mL, SD 0.05; *p* = 0.003). For those with current hypertension (≥140 and/or 90 mm Hg), urinary MC/NOD detection was more prevalent and higher compared to those without current hypertension (3/13, 23% vs. 0/26, 0%; *p* = 0.01) (mean 0.07 ng/mL, SD 0.14 vs. mean 0 ng/m; *p* = 0.01) (Figure 4). Urine MC/NOD also showed higher prevalence and higher levels among those with a history of hypercholesterolemia compared to those with no history (2/3, 66.7% vs. 0/29, 0%; *p* < 0.0001) (mean 0.15 ng/mL, SD 0.13 vs. mean 0 ng/mL; *p* < 0.0001). Salivary MC/NOD levels were higher among those who had consumed alcohol within the past month compared to those with no consumption (mean 0.27 ng/mL, SD 0.82 vs. mean 0.07 ng/mL, SD 0.09; *p* = 0.03).

## 4. Discussion

We report novel findings demonstrating that exposure to cyanobacteria hepatotoxins may be prevalent among residents of the U.S. Pacific Island territory of Guam. These findings are particularly compelling given the high incidence of liver cancer in Guam (world age-standardized incidence of 24.9 and 5.1 per 100,000 males and females, respectively) and other regions of Micronesia [15,16]. Notably, MC/NOD levels were specifically elevated in individuals with certain liver cancer risk factors [20]—hyperlipidemia and hypertension as well as alcohol consumption. Our study also provides indirect evidence suggesting that exposure to cyanotoxins may originate from environmental sources including drinking water and locally-grown produce.

There is limited knowledge regarding the pharmacokinetics of cyanotoxins in humans including their production, absorption, uptake, distribution, metabolism, and excretion. As we did not evaluate liver samples nor directly measure in situ production and metabolism of cyanotoxins, our ability to ascertain pharmacokinetics in the present study is limited. Largely based on in vitro and in vivo studies of MC, we can generally postulate that oral cyanotoxins enter the gastrointestinal system where they are absorbed into the blood stream and are taken up into the liver via OATP transporters [21]. From the liver, cyanotoxins are excreted in bile through which they can recirculate back to the liver via enterohepatic recirculation and from which they can enter the bloodstream to access the kidney and intestines where they are excreted in urine and feces, respectively. In our prior work, we detected both cyanobacteria and cyanotoxins in oral swabbings and saliva samples [17]. Accordingly, cyanotoxins detected in the present study were likely both ingested from environmental sources and produced de novo from oral cyanobacteria. Nonetheless, we did not assess oral cyanobacteria in samples, which limited our ability to fully assess oral exposure. We show for the first time that MC/NOD, CYN, and AB are variably detected in saliva, serum, and urine. Overall, MC/NOD prevalence was highest in serum, CYN in urine, and AB in both serum and urine. MC/NOD levels were most variable with detection ranging from 7.5% in urine to 100% in serum. The low prevalence of urinary MC/NOD contrasts with detection of 85 and 100% for urinary AB and CYN, respectively. These differences likely reflect pharmacokinetic variation across cyanotoxins.

Evidence of human health effects of cyanotoxins have largely been limited to instances of acute MC exposure which have resulted in deaths and severe liver manifestations including severe inflammation and necrosis as well as abnormal liver enzymes and elevated bilirubin and triglycerides [22,23]. There is little knowledge of the health consequences of cyanotoxin exposure, particularly at chronic, low levels. To date, there is largely indirect evidence linking cyanotoxins to liver cancer in human populations. In China, populations consuming drinking water from pond and ditch surface water sources where cyanobacteria and MCs are abundant were shown to have elevated risks of incident HCC and HCC mortality [24,25,26]. In the U.S. and Europe, the geographic location of bodies of water containing cyanobacteria blooms resulting from cyanobacteria overgrowth have been linked to significant clusters of liver disease and liver cancer death [27,28,29]. To date, only one study has evaluated the association of cyanotoxin exposure with liver cancer risk. Serum MC-LR levels were found to be an independent risk factor for HCC in a hospital-based study of HCC cases and age/sex-matched controls in southwest China (adjusted OR 2.9, 95% CI 1.5–5.5), and risk increased with increasing levels of MC-LR [13]. Notably, serum MC-LR levels in both cases and controls were lower than that found in our study population. We recently showed that oral cyanobacteria may be an independent risk factor for the development of HCC in a northeast U.S. population [14]. Cyanobacteria was positively associated with HCC (odds ratio 8.71, 95% CI 1.22–62.00) after adjustment for age, race, birthplace, education, smoking, alcohol, obesity, type 2 diabetes, HCV, HBV, fatty liver disease, aspirin use, other NSAID use, laboratory batch, and other significant taxa. Cyanobacterial genes positively associated with HCC were specific to taxa producing MC and other genes known to be upregulated with MC exposure.

In the present study, we observed elevated urine MC/NOD levels in individuals with a history of hyperlipidemia and hypertension—conditions which, along with obesity and type 2 diabetes, characterize metabolic syndrome, which typically manifests as non-alcoholic fatty liver disease (NAFLD), an increasingly important precursor of liver cancer globally [30]. Specifically, urine MC/NOD levels were elevated in individuals with hypertension and hyperlipidemia, and salivary MC/NOD in those with recent alcohol consumption. Our findings are consistent with in vivo evidence that NAFLD can influence MC uptake and toxicity through the alteration of expression of hepatic OATPs, which are required for uptake of MC into the liver [31]. Notably, prior studies also suggest that chronic low dose cyanotoxin exposure can exacerbate the pathogenesis and progression of NAFLD characterized, in part, by increased plasma cholesterol, aberrant fatty acid β-oxidation and hepatic lipoprotein secretion, and increased hepatic inflammation and steatosis [31,32,33,34].

Guam’s high liver cancer incidence may reflect the contribution of established and unique risk factors prominent in the population, including obesity and type 2 diabetes [35]. Obesity, hypertension, and hyperlipidemia were prevalent in the study population. AN/BQ chewing, practiced by over half of study participants, has been identified as a potential risk factor for liver cancer [36,37]. Epidemiologic studies, primarily from Taiwan, have reported independent associations of betel nut chewing with liver cirrhosis and HCC after adjustment for sex, age, viral hepatitis, smoking, alcohol consumption, and other factors [38,39,40,41,42,43]. The mechanism underlying the potential relationship of AN/BQ to liver cancer is unclear. Habitual AN/BQ chewing typically involves chronic, daily exposure where the nut or quid is chewed for prolonged periods of time. Our study suggests that AN/BQ chewing in Guam may be a source of chronic oral exposure to hepatotoxins originating from cyanobacteria-contaminated AN/BQ plants.

Our assessment of cyanotoxins did not include water sources nor locally grown fruits and vegetables. Accordingly, our findings provide only limited, indirect evidence suggesting that local drinking water and local agricultural products are potential sources of cyanotoxin exposure. MC/NOD levels in both saliva and urine were higher among individuals using home tap water as the primary drinking water source compared to those using store-bought/commercial water. Urinary MC/NOD levels were elevated among individuals consuming vegetables and fruits entirely from locally-grown sources. This is consistent with primary routes of human exposure to cyanotoxins through ingestion of contaminated drinking water and contaminated food sources [11]. Cyanotoxins can occur in surface waters—including freshwater, brackish, and marine sources—as well as groundwater, which can lead to contamination of drinking water sources [12]. Soil and plants can become contaminated by groundwater as well as through irrigation with affected water. We did not find any differences in cyanotoxin levels with seafood consumption, which has been shown to be a potential source of exposure [44,45].

Our findings support the possibility that cyanotoxins may be widespread within Guam’s terrestrial environment with nearly half of plant samples containing one or more cyanotoxins. While cyanotoxin levels in locally-grown plant samples did not vary by island region, the variation by plant types may indicate some differences across sources. AB was found in over half of all plant samples, which is consistent with emerging evidence that this cyanotoxin is one of the most prevalent in the global environment [46]. AB has been shown to influence levels of other cyanotoxins by inducing lysis of certain cyanobacteria and subsequent release of cyanotoxins [47]. This is supported by the observed strong correlation of AB levels with other cyanotoxins in both plant and human samples. Notably, AB levels in plant and human samples appeared to reflect exposures outside of Guam with elevated levels in Yap AN/BQ plant samples, higher urinary AB levels in individuals consuming imported vegetables, and detection of serum AB in all individuals born in Pacific Island jurisdictions outside of Guam. Cyanobacteria and cyanotoxins have been previously documented from both terrestrial and aquatic environments in Guam. The cyanobacteria neurotoxin β-methylamino-l-alanine (BMAA) was isolated from the roots and seeds of *Cycas micronesica* trees native to Guam [48]. Cyanobacteria blooms have been observed in Guam’s coral reefs [49]. The marine cyanobacteria, Lyngbya, has been identified in Guam’s reef and lagoon habitats [50]. Marine and terrestrial cyanobacteria may have overlapping habitats in Guam where ocean species have been observed to wash up and affect surrounding land areas [51].

The clinical significance of our findings is unclear as our investigation did not focus on individuals with liver cancer or chronic liver disease. This cross-sectional study design and small study population of volunteers recruited from the community limits the validity and generalizability of our study findings. Comparisons of cyanotoxin detection across saliva, urine, and blood samples were also hampered by the variable donation of different biospecimens including urine, which was collected for only half of participants. The structural similarity of MC and NOD did not allow us to distinguish these two cyanotoxins in the ELISA assay. Nonetheless, we can presume MC to be the predominant analyte, as it is one of the most common cyanotoxins produced by multiple cyanobacteria species while NOD-producing species are comparatively limited [4]. While ELISA is widely used for cyanotoxin assessment, the inclusion of other modes of detection such as liquid chromatography-mass spectrometry (LC-MS) and high performance liquid chromatography (HPLC) would have allowed for confirmation of ELISA results as well as identification of cyanotoxin variants. We were limited in our ability to characterize environmental cyanotoxins based on the small number of plant samples assessed. Drinking water, freshwater and ocean water sources, seafood, and other agricultural produce were not evaluated, which limited our ability to fully ascertain the extent of environmental sources. Furthermore, we were not able to distinguish potential exposure through oral, dermal, and aerosolized routes.

Our study provides evidence suggesting that exposure to cyanobacterial hepatotoxins, including tumor promoters, may be prevalent in Guam and may originate from environmental sources. Cyanobacteria and cyanotoxins remain largely unregulated and unmonitored in most jurisdictions, including Guam and the United States. The World Health Organization has issued provisional guidelines for human exposure to drinking water and recreational sources [52]. A number of U.S. states also have developed surveillance systems for monitoring and reporting suspected cyanobacteria-contaminated water bodies including drinking water and recreational water sources. Research is needed to address the paucity of information on the health consequences of cyanotoxin exposure within communities worldwide. Importantly, population-based epidemiologic studies are needed to investigate the role of cyanotoxins in liver cancer development.

## Figures and Tables

**Table 1:** Study Population Characteristics (*n* = 73).

	No.	%
***Biospecimen collection***		
Blood	54	74.0
Saliva	65	89.0
Urine	40	55.0
***Age (years) (mean 34.8, range 20–64)***		
20–39	54	74.0
≥40+	19	26.0
***Sex***		
Male	34	46.6
Female	39	53.4
***Race/ethnicity***		
Chamoru	30	41.1
Chuukese	22	30.1
Other	21	28.8
***Education level***		
High school	13	20.0
college graduate 5	28	43.1
Post-graduate	24	36.9
***Marital status***		
Single	46	64.8
Ever married	25	35.2
***AN/BQ chewing status***		
Current	35	50.7
Former	10	14.5
Never	24	34.8
***Cigarette smoking daily***		
Yes	11	15.3
No	61	84.7
***Alcohol consumption in past 30 days***		
Yes	42	61.8
No	26	38.2
***Obese (BMI ≥30)***		
Yes	43	61.4
No	27	38.6
***History of high cholesterol***		
Yes	12	19.7
No	49	80.3
***History of type 2 diabetes***		
Yes	6	8.8
No	62	91.2
***History of hypertension***		
Yes	19	29.7
No	45	70.3
***Measured hypertension***		
Yes	28	40
No	42	60
***History of non-alcoholic fatty liver disease***		
Yes	2	2.9
No	67	97.1
***Primary drinking water source***		
Home faucet-filtered	11	16.4
Bottled/Store-bought	53	79.1
Rain water	3	4.5
***Fruit source***		
Locally-grown	5	9.1
Imported	19	34.5
Locally-grown and imported	31	56.4
***Vegetable source***		
Locally-grown	5	8.8
Imported	21	36.8
Locally-grown and imported	31	54.4
***Freshwater seafood consumption***		
Never or rarely	37	56.1
Some days	28	42.4
Every day	1	1.5
***Ocean water seafood consumption***		
Never or rarely	25	37.9
Some days	39	59.1
Every day	2	3
***Frequency of water recreational activity***		
Never	16	26.2
Weekly	5	8.2
Monthly	12	19.7
Occasionally	28	45.9

Missing data: Education (*n* = 8); Marital status (*n* = 2); AN/BQ chewing (*n* = 4); Cigarette smoking (*n* = 1); Alcohol (*n* = 5); Obese (*n* = 3); Cholesterol (*n* = 12); Diabetes (*n* = 5); Hypertension (*n* = 9); Measured hypertension (*n* = 3); NAFLD (*n* = 4); Water source (*n* = 6); Fruit source (*n* = 18); Vegetable source (*n* = 16); Freshwater seafood (*n* = 7); Ocean seafood (*n* = 7); Water recreation (*n* = 12).

## Data Availability

Data will be made available upon request.
